# Pulmonary endometriosis: a rare cause of hydropneumothorax

**DOI:** 10.1002/rcr2.432

**Published:** 2019-05-20

**Authors:** Husam Alzayer

**Affiliations:** ^1^ Department of Internal Medicine Galway University Hospitals Galway Ireland

**Keywords:** Catamenial hydropneumothorax, endometriosis, hydropneumothorax, pulmonary endometriosis, tuberculosis

## Abstract

Pulmonary involvement in endometriosis is well described in the literature but asymptomatic significant hydropneumothorax is considered an unusual presentation. It classically coincides with a menstrual cycle and can be a cause of recurrent disease. We present a young lady who was found to have an incidental asymptomatic right hydropneumothorax on a pre‐employment health screen. She comes from an endemic area of tuberculosis, thus we pursued several diagnostic tests to rule it out. A diagnosis of catamenial hydropneumothorax was reached through a pleural biopsy. This case highlights the importance of adopting a systematic approach in managing uncommon presentations.

## Introduction

Pulmonary involvement in endometriosis is well described in the literature but asymptomatic significant hydropneumothorax is considered an unusual presentation 1. It classically coincides with a menstrual cycle 2 and can be a cause of recurrent disease. We present a rare case of catamenial hydropneumothorax confirmed through a pleural biopsy.

## Case Report

**Figure 1:**
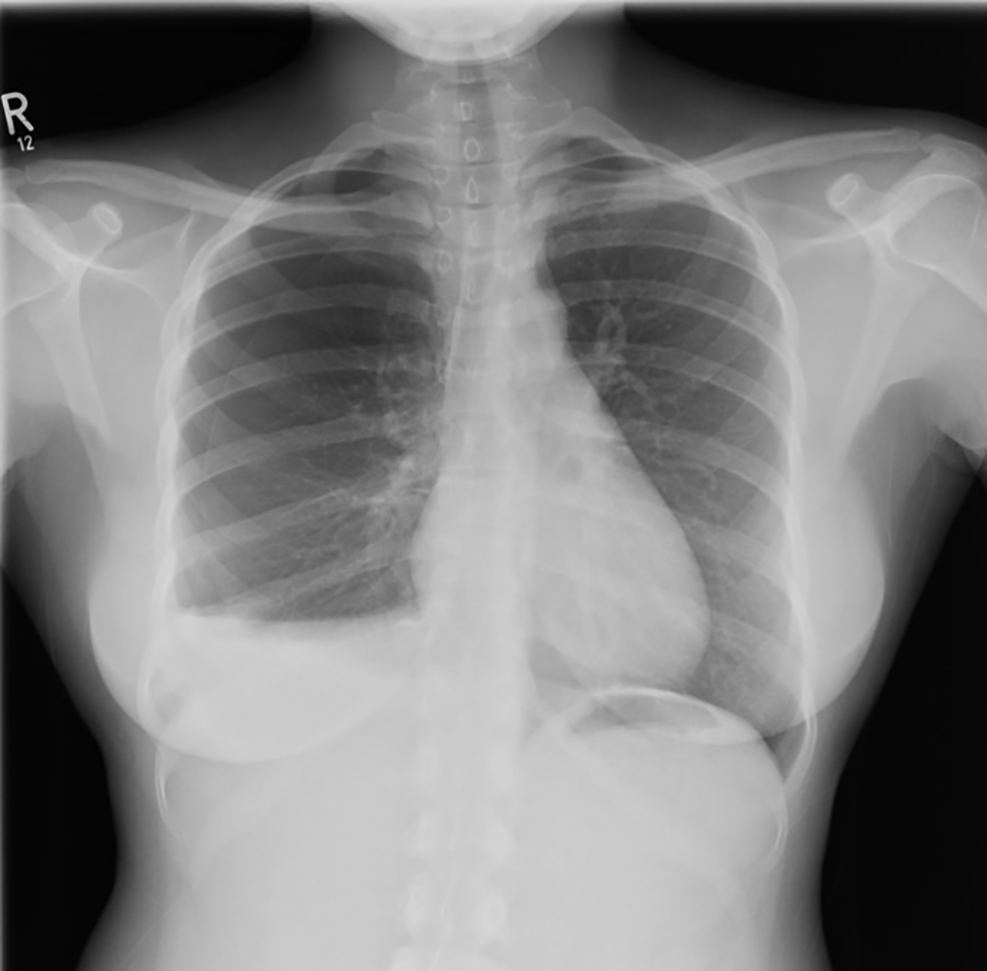
Chest X‐ray showing a right hydropneumothorax with clear lung fields and no mediastinal fullness.

A 30‐year‐old African nurse was referred to the Accident & Emergency after an incidental finding of an asymptomatic right hydropneumothorax on a chest X‐ray (Fig. 1) as part of her occupational health screen. She has been in Ireland for two months with a reported normal chest X‐ray done prior to her arrival. She denied any constitutional or respiratory symptoms with no recent trauma and unremarkable past medical and surgical history and no use of regular medications. She was a non‐smoker with up‐to‐date vaccination status including Bacillus Calmette–Guérin vaccine scar and no known contact with tuberculosis patients. Her examination revealed absent right apical breath sounds but otherwise unremarkable with no lymphadenopathy.

**Figure 2:**
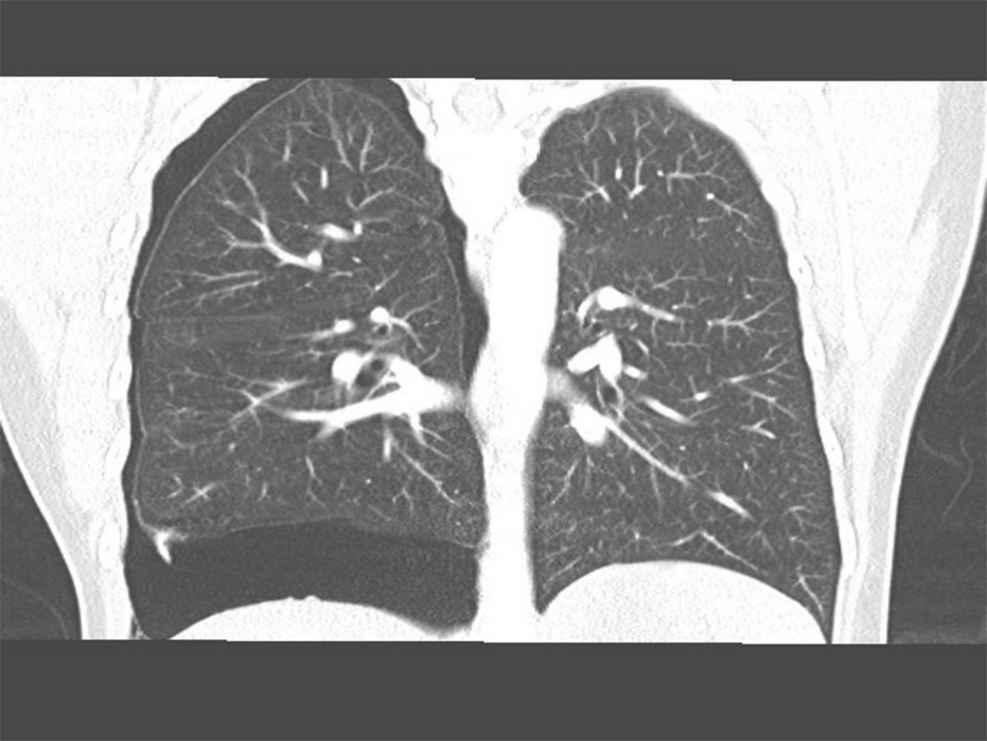
Coronal plane of computed tomography chest showing large right pneumothorax occupying approximately 20% of right hemithorax.

**Figure 3:**
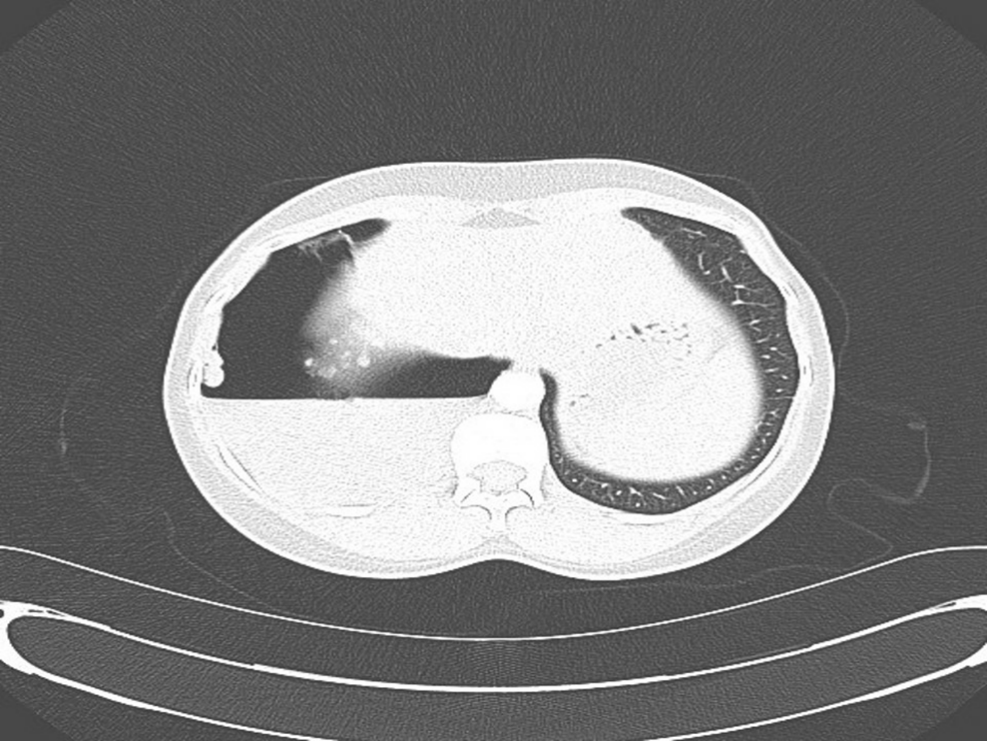
Transverse plane of computed tomography chest showing more clearly unilocular‐dependent fluid with large right hydropneumothorax.

Initial investigations showed a mild microcytic hypochromic anaemia. Her C‐reactive protein was normal and had a negative viral screen, normal liver enzymes, and normal urea and electrolytes. A computed tomography thorax was done for further evaluation, described large right hydropneumothorax occupying approximately 20% of right hemithorax with unilocular‐dependent fluid along with multiple pleural tags and 1 cm right pleural nodule (Figs. 2,3). A chest tube was inserted draining blood‐stained fluid, which came back exudative, that was sent for staining and cultures.

The differential diagnosis of an exudative effusion is broad 3, 4; however, as tuberculosis is endemic in Africa, it remained a concern. Other differentials included vascular, autoimmune, malignancy, and genitourinary.

**Figure 4:**
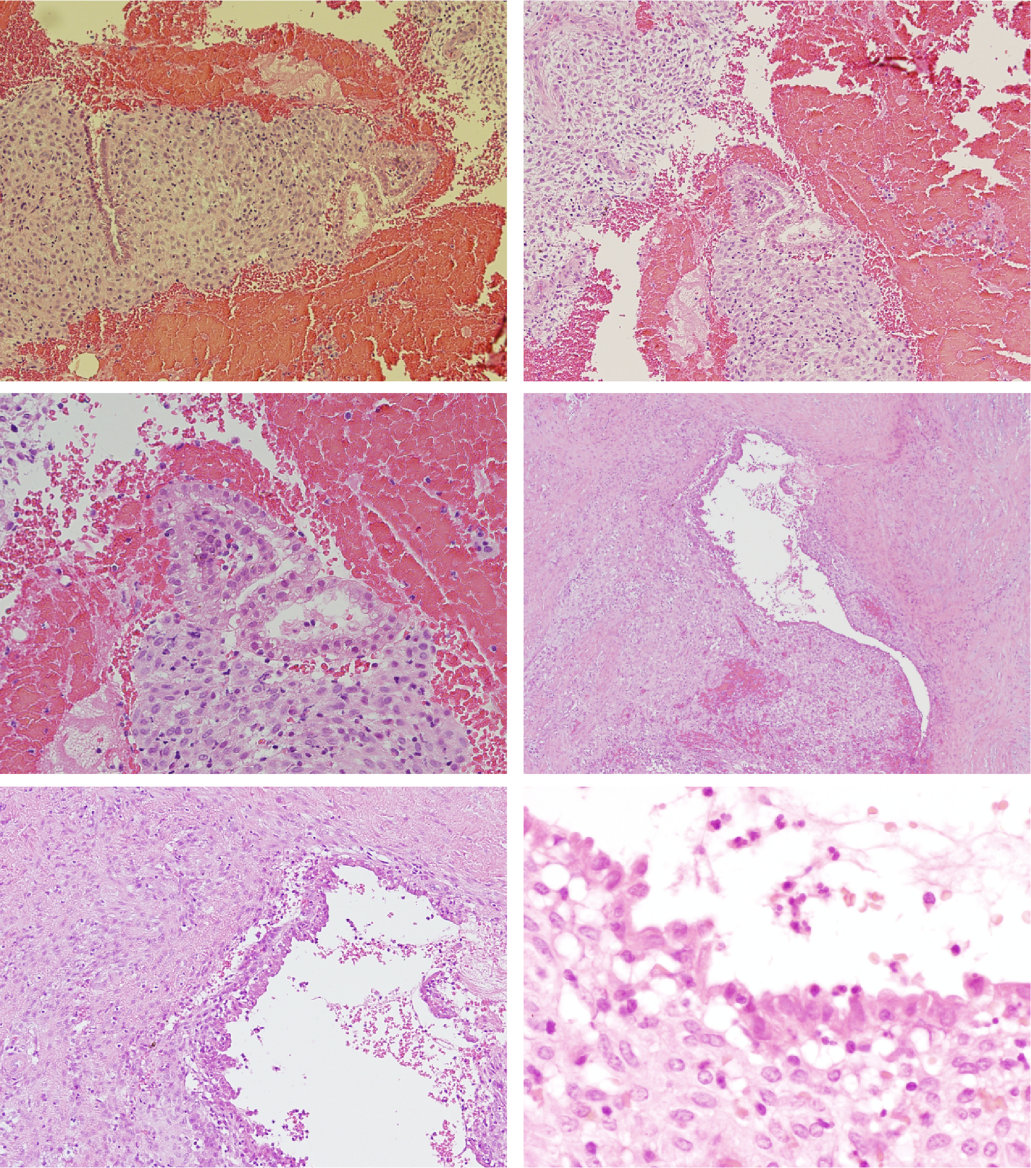
Fibroconnective tissue and skeletal muscle show the presence of benign endometrial glands and stroma (interval phase) with associated chronic inflammation at various powers (10×, 20×, and 40×). This was further confirmed with positive estrogen receptor (ER) and cluster of differentiation (CD)10 staining.

A final diagnosis of catamenial hydropneumothorax was reached based on the histological findings from a pleural biopsy (Fig. 4); it showed benign endometrial glands and chronic inflammation. No malignancy or granulomatous inflammation was identified. There was no evidence of an infectious process. She had normal inflammatory markers, negative cultures, and negative tuberculosis polymerase chain reaction and culture.

She was kept on the chest drain for a total of 14 days until complete resolution of her hydropneumothorax and was commenced on combined oral contraceptive pill, which contains ethinylestradiol and levonorgestrel, and was discharged with a close follow‐up with the respiratory and gynaecology outpatient service.

## Discussion

Endometriosis is characterized by the presence of endometrial‐like tissue outside the uterus and is thought to affect 6%–10% of women of childbearing age 5 and thoracic endometriosis incidence in the general population is unknown. Catamenial pneumothorax and pleural effusion are well described extra pelvic manifestations of endometriosis but extremely rare to present together 1 and can happen in the absence of pelvic involvement. Other forms of thoracic involvement presentations include catamenial haemoptysis, endometriotic lung nodules, and catamenial chest pain 6. There are certain risk factors for developing endometriosis such as nulliparity, early menarche/late menopause, short menstrual cycles, prolonged menses, and tall thin body habitus but it is unclear if they also increase the risk for pulmonary involvement.

This case highlights the importance of a systematic diagnostic approach to establish the correct diagnosis despite unusual presentation. A diagnostic approach of pleural effusion and pneumothorax was made based on transudative versus exudative 3 and primary versus secondary 4, respectively. As the patient came from a country with a high burden of tuberculosis, this was the primary differential. Seeking a tissue biopsy is essential to reach a diagnosis of pulmonary endometriosis 7 and is the highest yield when suspecting pleural tuberculosis.

Adopting a systematic approach in managing uncommon presentations aids in establishing a diagnosis including seeking a tissue biopsy. Pneumothorax coinciding with a menstrual period should alert clinicians to the possibility of catamenial pulmonary disease.

The management approach in catamenial hydropneumothorax is to start with definitive treatment by proper drainage through a large bore chest tube followed by secondary prevention as there is high rate of reoccurrence. This is recommended with surgical blebectomy, pleurodesis, or diaphragmatic repair 8 followed by hormonal suppressive therapy for 6–12 months and can be extended if appropriate to reduce the risk of reoccurrence. A gonadotropin‐releasing hormone analogues or combined oestrogen and progesterone contraceptive medication, with better results on the former, can be used depending on patient’s tolerability of the side effects 9.

### Disclosure Statement

Appropriate written informed consent was obtained for publication of this case report and accompanying images.
